# Genotypic Diversity of Ciprofloxacin Nonsusceptibility and Its Relationship with Minimum Inhibitory Concentrations in Nontyphoidal *Salmonella* Clinical Isolates in Taiwan

**DOI:** 10.3390/antibiotics10111383

**Published:** 2021-11-11

**Authors:** Shiuh-Bin Fang, Tsai-Ling Yang Lauderdale, Chih-Hung Huang, Pei-Ru Chang, Yuan-Hung Wang, Katsumi Shigemura, Ying-Hsiu Lin, Wei-Chiao Chang, Ke-Chuan Wang, Tzu-Wen Huang, Yu-Chu Chang

**Affiliations:** 1 Division of Pediatric Gastroenterology and Hepatology, Department of Pediatrics, Shuang Ho Hospital, Taipei Medical University, New Taipei City 23561, Taiwan; claire7741@tmu.edu.tw (P.-R.C.); noble019@tmu.edu.tw (Y.-H.L.); d102094010@tmu.edu.tw (K.-C.W.); 2 Department of Medical Research, Shuang Ho Hospital, Taipei Medical University, New Taipei City 23561, Taiwan; d508091002@tmu.edu.tw; 3 Department of Pediatrics, School of Medicine, College of Medicine, Taipei Medical University, Taipei 11031, Taiwan; 4 Master Program in Clinical Pharmacogenomics and Pharmacoproteomics, College of Pharmacy, Taipei Medical University, Taipei 11031, Taiwan; wcc@tmu.edu.tw; 5 National Institute of Infectious Diseases and Vaccinology, National Health Research Institutes, Zhunan 35053, Taiwan; lauderdale@nhri.org.tw; 6 Graduate Institute of Biochemical and Biomedical Engineering, National Taipei University of Technology, Taipei 10608, Taiwan; chhuang@ntut.edu.tw; 7 Graduate Institute of Clinical Medicine, College of Medicine, Taipei Medical University, Taipei 11031, Taiwan; 8 Department of Urology, Kobe University Graduate School of Medicine, Kobe 650-0017, Japan; katsumi@med.kobe-u.ac.jp; 9 Center for Hyperpolarization in Magnetic Resonance, Department of Health Technology, Technical University of Denmark, DK-2800 Kongens Lyngby, Denmark; 10 Department of Microbiology and Immunology, School of Medicine, College of Medicine, Taipei Medical University, Taipei 11031, Taiwan; tw.huang@tmu.edu.tw; 11 Department of Biochemistry and Molecular Cell Biology, School of Medicine, College of Medicine, Taipei Medical University, Taipei 11031, Taiwan; yuchuc@tmu.edu.tw

**Keywords:** ciprofloxacin nonsusceptibility, minimum inhibitory concentrations, quinolone resistance determining regions, plasmid-mediated quinolone resistance, nontyphoidal *Salmonella*

## Abstract

This study analyzed the genetic diversity of ciprofloxacin (CIP) nonsusceptibility and the relationship between two major mechanisms and minimum inhibitory concentrations (MICs) of CIP in nontyphoidal *Salmonella* (NTS). Chromosomal mutations in quinolone resistance-determining regions (QRDRs) and plasmid-mediated quinolone resistance (PMQR) genes were searched from ResFinder, ARG-ANNOT, and PubMed for designing the sequencing regions in *gyrA*, *gyrB*, *parC*, and *parE*, and the 13 polymerase chain reactions for PMQR genes. We found that QRDR mutations were detected in *gyrA* (82.1%), *parC* (59.0%), and *parE* (20.5%) but not in *gyrB* among the 39 isolates. Five of the 13 PMQR genes were identified, including *oqxA* (28.2%), *oqxB* (28.2%), *qnrS* (18.0%), *aac*(6′)-*Ib*-*cr* (10.3%), and *qnrB* (5.1%), which correlated with the MICs of CIP within 0.25–2 μg/mL, and it was found that *oxqAB* contributed more than *qnr* genes to increase the MICs. All the isolates contained either QRDR mutations (53.8%), PMQR genes (15.4%), or both (30.8%). QRDR mutations (84.6%) were more commonly detected than PMQR genes (46.2%). QRDR mutation numbers were significantly associated with MICs (*p* < 0.001). Double mutations in *gyrA* and *parC* determined high CIP resistance (MICs ≥ 4 μg/mL). PMQR genes contributed to intermediate to low CIP resistance (MICs 0.25–2 μg/mL), thus providing insights into mechanisms underlying CIP resistance.

## 1. Introduction

In 2017, the World Health Organization listed fluoroquinolone (FQ)-resistant *Salmonella* spp. as priority 2 (high) pathogens for which novel antibiotics are urgently required [1]. The resistance of nontyphoidal *Salmonella* (NTS) to ciprofloxacin (CIP) has been increasing worldwide for the past two decades [2,3,4,5,6,7,8]. CIP is one of the most commonly prescribed FQs as the second-line antibiotic for medical use when narrow-spectrum antibiotics are ineffective [9]. However, one or a combination of mutations within quinolone resistance–determining regions (QRDRs) can cause FQ resistance either by changing the drug-binding affinity of two bacterial type II topoisomerases, namely DNA gyrase (encoded by *gyrA* and *gyrB*) and DNA topoisomerase IV (encoded by *parC* and *parE*), or by reducing the intracellular drug concentration through either decreased uptake or increased efflux; in addition, FQ resistance can occur due to the production of drug-modifying enzymes, target-protection proteins, or efflux pumps by plasmid-mediated quinolone resistance (PMQR) genes [9,10,11]. These molecular mechanisms are not mutually exclusive and can be accumulative.

Genotypic features of CIP nonsusceptibility caused by QRDR mutations and PMQR genes in NTS human isolates can vary with time and country. In a large survey conducted in Taiwan during 1999–2008, four PMQR genes *oqxAB* (16.1%), *qnrS* (4.8%), *qnrD* (3.2%), and *aac*(6′)*-Ib-cr* (1.6%) were identified as resulting in CIP nonsusceptibility. High quinolone resistance could be attributable to *gyrA* mutations Ser83Phe/Asp87Asn (80.6%) and Ser83Phe/Asp87Gly (16.7%) [12]. A large clinical survey in Spain during 2004–2008 revealed *gyrA* mutations (mainly Asp87 and Ser83 substitutions) in 80% and *parC* mutations in 5% (Thr57 substitution) of 105 human CIP-nonsusceptible NTS isolates, with only one strain carrying *qnrS1* without QRDR mutations [3]. Another study conducted in Switzerland during 2005–2011 reported the substitution of Ser83Phe in *gyrA* and Ser80Ile in *parC* in all 16 CIP-resistant *Salmonella* human isolates, but PMQR genes were detected only in four CIP-intermediate strains [13]. Several recent studies have reported an association of PMQR genes with the CIP nonsusceptibility of NTS isolates [11,14,15,16,17]. In Ghana during 2016–2018, *qnrS* was found in two of five CIP-intermediate NTS human isolates harboring *gyrA* mutation in Ile203Ser [14]. A study in the United States during 2008–2014 detected *qnrB* (61.1%), *qnrS* (27.8%), *qnrA* (5.6%), and *aac*(6′)*-Ib-cr* (4.2%) in 24% of NTS human isolates with a minimum inhibitory concentration (MIC) of CIP of >0.25 μg/mL and susceptibility to nalidixic acid [15]. By contrast, *gyrA* and *qnrA* mutations were noted in 95.2% and only 4.8%, respectively, of CIP-nonsusceptible NTS isolates obtained from Korean patients in 2016 [16]. However, *qnrS* was the most common PMQR gene identified in a recent Korean study that reported a single PMQR gene (*qnrA*, *qnrB*, or *qnrS*) and two PMQR genes (*qnrS* and *aac*(6′)*-Ib-cr* or *qnrA* and *qnrB*) present in 64.7% and 8.8% of CIP-nonsusceptible *Salmonella* strains, respectively [17]. Thus far, QRDR mutations in *gyrA* and *parC* have been more frequently observed than those in *gyrB* and *parE*; however, PMQR genes additively contribute to FQ resistance with considerably varying incidences [11]. How interplay occurs between multiple mechanisms in FQ resistance remains obscure [9].

In this study, we investigated the presence of QRDR mutations and PMQR genes through molecular biology in CIP-nonsusceptible NTS clinical isolates representatively sampled from different regions of Taiwan, and analyzed the prevalence of the detected genetic loci and their relationship with MICs.

## 2. Results

### 2.1. Concomitant Resistance to Ampicillin and Ceftriaxone in the CIP-Nonsusceptible NTS Isolates

In our study, 34 (87%) and 2 (5.1%) of the 39 CIP-nonsusceptible isolates were resistant to ampicillin and ceftriaxone, respectively.

### 2.2. Detected Genomic Point Mutations in Three QRDR Genes

A total of nine reported mutations in the QRDR with eight amino acid substitutions (codons 248, 259, and 260 in *gyrA*; codons 170, 238, 239, and codon 250 in *parC*, and codon 1372 in *parE*) were detected in the 39 CIP-nonsusceptible NTS isolates (Table 1). Among the 39 NTS isolates, QRDR mutations were present in *gyrA* of the 32 (82.1%) isolates, *parC* of the 23 (59.0%) isolates, and *parE* of the 8 (20.5%) isolates but not in *gyrB* of any isolate. No QRDR mutation or PMQR gene was present in more than 50% of the CIP-nonsusceptible NTS isolates except for two QRDR mutations, namely Thr57Ser in *parC* (58.9%) and Ser83Phe in *gyrA* (53.8%). Other reported QRDR mutations in *gyrA*, *gyrB*, and *parC* in Table S1 were not detected.

### 2.3. Detected Five PMQR Genes

A total of five known PMQR genes were identified in the 39 CIP-nonsusceptible NTS isolates (Table 1), namely *aac*(6′)-*Ib*-*cr* (10.3%; Supplementary Materials Figure S1A), *oqxA* (28.2%; Figure S1B) and *oqxB* (28.2%; Figure S1C) simultaneously and *qnrB* (5.1%; Figure S1D), and *qnrS* (18.0%; Figure S1E). The other eight PMQR genes, namely *qepA*, *qnrA*, *qnrC*, *qnrD*, *qnrAS*, *qnrSM*, *qnrVP*, and *qnrVV*, were not detected in all the 39 CIP-nonsusceptible NTS isolates (Figure S2).

### 2.4. Distribution of Detected QRDR Mutations and PMQR Genes

**Figure 1:**
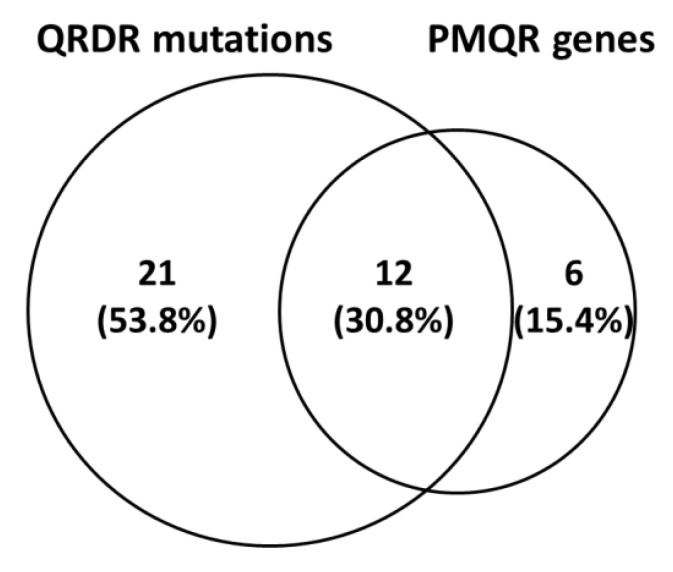
Venn diaphragm of QRDR mutations and PRQR genes in the 39 CIP-nonsusceptible NTS isolates.

Of the 39 CIP-nonsusceptible NTS isolates, we observed that 12 (30.8%) isolates contained both QRDR mutations and PMQR genes, 21 (53.8%) isolates contained QRDR mutations only, and 6 (15.4%) isolates contained PMQR genes only (Figure 1). All the isolates contained either the reported QRDR mutations or known PMQR genes. Any QRDR mutation was detected in 33 (84.6%) of the 39 isolates, whereas any PMQR gene was noted in 18 (46.2%) of the 39 isolates (Table S2).

### 2.5. Relationship between Genetic Mechanisms and the MIC of CIP

In Group 1, a total of eight (20.5%) isolates with a high MIC of 32 μg/mL exhibited double QRDR mutations individually in *gyrA* and *parC*, and a single mutation in *pare*. In Group 2, 10 (25.6%) isolates with an MIC of 4–16 μg/mL had double QRDR mutations individually in *gyrA* and *parC* (Table 2). In Group 3, most of the 14 (35.9%) isolates with an MIC of 8 μg/mL had a single QRDR mutation in *gyrA* or/and in *parC* together with PMQR genes, except for three isolates in Types VI and IX that harbored only PMQR genes. In Group 4, seven (17.9%) isolates with an MIC of 0.25–0.5 μg/mL had a single QRDR mutation in *gyrA* or/and *parC*, presence of PMQR genes alone, or a single QRDR mutation in *parC* with *qnrB*.

Without QRDR mutations, the presence of either of the three PMQR genes resulted in higher CIP resistance in the two isolates with an MIC of 2 μg/mL in Type VI compared with the presence of one or two PMQR genes in the other four isolates with MICs of 0.25–1 μg/mL in Types IX, XII, and XI (Table 2). The PMQR gene *aac*(6′)-*Ib*-*cr* was present only in three CIP-intermediate isolates in Types XI and XII, whereas another PMQR gene *qnrB* was present in only two CIP-intermediate isolates in Types XI and XIV (Group 4, Table 2). The other PMQR, gene *qnrS*, was detected in the seven isolates with MICs of 0.25–2 μg/mL.

The 39 collected clinical isolates were classified into four groups according to four different ranges of MICs (Table 3), and also classified into three groups according to QRDR mutation numbers (5, 4, and 0–3 mutations). Statistical analysis showed significantly positive associations between three groups of QRDR mutation numbers and four ranges of MICs (*p* < 0.001, Table 4).

## 3. Discussion

CIP nonsusceptibility can more accurately reflect the genuine clinical situation than CIP resistance in FQ-treated patients with salmonellosis. A study conducted in 2003 reported that both Typhi and non-Typhi *Salmonella* isolates exhibited resistance to nalidixic acid with decreased susceptibility and clinical response to FQs [18]. Before 2012, all Enterobacteriaceae shared common MIC and disk diffusion breakpoints for different FQs in the CLSI 2011. However, the CLSI M100 2012 re-evaluated the interpretive criteria for the susceptibility of extraintestinal *Salmonella* isolates to CIP and adopted new *Salmonella*-specific breakpoints as used in the Table 5 of this study. This information facilitates clinicians in deciding the maximal dosage and duration of FQs, or prescribing alternative antibiotics for patients infected by CIP-intermediate isolates [19]. CIP nonsusceptibility should be carefully evaluated because even a minor increase in the MIC of quinolone could unfavorably affect the treatment response [18,20,21]. In our study, the percentage of the CIP-nonsusceptible NTS isolates with ampicillin resistance (87%) was higher than that reported in a study conducted in Ethiopia (58.6%) [22]. Altogether, CIP nonsusceptibility and its increased co-resistance to other antibiotics indicate the worsening problems of treatment failure and delayed clinical responses in *Salmonella*.

The random detection or sequencing of hotspot genes limits the investigation in the genotypic diversity of genetic loci associated with CIP nonsusceptibility in NTS. The location and number of QRDR mutations and PMQR genes may contribute to the intensity of CIP resistance that is reflected by a quantitative change in the MIC. Therefore, we classified our 39 CIP-nonsusceptible NTS isolates into four groups, according to ranges of MICs, and 14 types, based on the combination of QRDR mutations and PMQR genes. Our grouping analysis demonstrated that *gyrA* and *parC*, present in 21 (53.8%) of the 39 isolates, were the major QRDR genes with genomic mutations accounting for CIP nonsusceptibility, particularly leading to high MICs. These two QRDR genes, harboring at least two mutations, resulted in a high MIC of ≥4 μg/mL for CIP, and 17 of the 18 (95%) isolates had an MIC of ≥8 μg/mL. In contrast, only intermediate resistance to CIP was observed in isolates harboring a single mutation in *gyrA* and *parC* individually and a single mutation in *gyrA* (Types X and XIII). The other QRDR gene, *parE,* exerted an additive synergistic effect on CIP resistance with *gyrA* and *parC*. A single mutation in *parE* led to a four-fold increase in the MIC by up to 32 μg/mL, compared with the isolate that harbored double mutations individually in *gyrA* and *parC* (Type I vs. Type IV). However, the add-on effect of PMQR genes on *gyrA* in elevating MICs of CIP was not as strong as that on *parC* and *parE*. When only one single mutation was individually present in *gyrA* and *parC* (Type X), an additional effect of *qnrS* (Type VII) increased the MIC of CIP by two-fold, thus increasing the level of resistance from intermediate to high. The additional effect of two and three PMQR genes increased the MICs of CIP to 1–2 μg/mL from 0.25 μg/mL when compared with only one single mutation in *gyrA* (Types VIII and V vs. Type XIII). The effect of CIP nonsusceptibility caused by *parC* was weaker than that caused by *gyrA* despite the coexistence of one *qnr* gene (Type XIV vs. Type VII). Furthermore, PMQR genes (*oqxA*, *oqxB*, *qnrS*, and *aac*(6′)-*Ib*-*cr*) exerted an additive synergistic effect on increasing CIP nonsusceptibility to resistance when only one single QRDR mutation was present in *gyrA* and/or *parC* (Type X vs. Types V, VII, and VIII). Furthermore, *aac*(6′)-*Ib*-*cr* exerted a cumulative effect on that of *oqxA* and *oqxB* in CIP resistance (Type V vs. VIII; Table 2). The effect of different resistance mechanisms on susceptibility to CIP based on data from *Escherichia coli* indicated that two *gyrA* mutations and one *parC* mutation caused a 60-fold change in the MIC of CIP, and one *gyrA* mutation caused a 10–16-fold change in the MIC of CIP; however, one *parC* mutation did not increase the MIC of CIP [9]. Our results showed a similar effect of simultaneous mutations in *gyrA* and *parC* on the MIC of CIP, but mutations in *parC* alone induced CIP nonsusceptibility. Furthermore, the presence of PMQR genes increased the MICs of CIP in the descending order of *qnr* (>30-fold change), *oxqAB* (16-fold change), and *aac*(6′)-*Ib*-*cr* (4-fold change) in *E. coli* [9]. Unlike *E. coli*, our five detected PMQR genes in NTS did not show a large difference in their CIP MICs between 0.25 and 2 μg/mL, and *oxqAB* contributed more than *qnr* genes to increasing CIP MICs. PMQR genes alone generally confer only low-level CIP nonsusceptibility compared with QRDR mutations.

The prevalence, number, and genomic loci of mutations in QRDR genes were correlated with their MICs. QRDR mutations in *gyrA* (82.1%) were more frequently observed than those in *parC* (59%) or *parE* (20.5%) in our 39 CIP-nonsusceptible NTS human isolates, and concurrent double mutations in both *gyrA* and *parC* coexisted in 18 strains (50%) highly resistant to CIP (Table 2). After in vitro exposure to FQs, compared with *parC*, *gyrA* was more inclined to undergo mutation in *Salmonella* spp., with the most frequent mutations observed in Asp87Asn and Asp87Tyr [23]. In addition, the predominance of *gyrA* with a rare report of *gyrB* was observed in other studies; however, the prevalence of *parC* and *parE* varied in human CIP-nonsusceptible NTS isolates. In our study, the most prevalent mutation in *gyrA* was Ser83Phe, followed by Ser83Tyr, Asp87Asn, and Asp87Gly (Table 1). This study and previous studies using human *Salmonella* isolates from Spain [3], Africa [8], Korea [24], and Taiwan [12,25] have consistently demonstrated mutations in *gyrA* as the leading determinant of FQ nonsusceptibility with Ser83 and Asp87 being the major hotspots, followed by commonly found mutations in *parC* and uncommonly found mutations in *gyrB* and *parE*. Each of the *gyrB* and *parC* mutants were rarely found in Africa [8]. Mutations in *parC* were detected at Thr57Ser [3,24], Ser80Arg/Ser80Ile [25], Thr57Ser, or Gly72Cys [24], whereas mutations were found in *gyrB* at Ser463Ala [22], Gly434Leu, or Gly447Cys, and in *parE* at Glu459Thr, Arg507Ile, or Lys514Asn [24]. One of the commonly detected mutations in *parC* at Thr57Ser was detected in *Salmonella* strains obtained from Finnish travelers without mutations in *gyrA*, *gyrB*, or *parE* [26]. The mutation Tyr57Ser in *parC* was also detected in 29 isolates with an MIC of >0.06 μg/mL in Hong Kong. Isolates with a single *gyrA* mutation were less resistant to FQs than those with an additional *parC* mutation (Tyr57Ser or Ser80Arg) [27]. In our study, Thr57Ser was the most prevalent mutation of *parC* in all the 23 strains, with their MICs increased to 4–32 μg/mL when a second *parC* mutation with double *gyrA* mutations coexisted. In accordance with a recent study, the *parC* mutation at Thr57Ser was detected in *Salmonella* pork isolates with the lowest (0.008–0.06 μg/mL) and highest MICs (0.025–2 μg/mL) of CIP being dependent on the type of *gyrA* mutation; high resistance to CIP (MIC: 32–64 μg/mL) was noted in all strains harboring multiple mutations in both *gyrA* and *parC* [28]. Accumulation of topoisomerase mutations leads to stepwise increases in resistance in *S. enterica* species, from mutations in GyrA at codons Ser83 and Asp87 to additional mutations in the same or a different target enzyme; other mechanisms (e.g., increased efflux or presence of PMQR genes) can result in high resistance levels [29,30].

The maximum diversity of PMQR genes depends on the number of PMQR genes selected for PCR in collected *Salmonella* isolates. To date, the detection of PMQR mechanisms usually requires up to six PCRs [21]. To detect the number of PMQR mechanisms, recent studies conducted in Korea, Taiwan, and the United States identified PMQR genes in human salmonellosis by performing five [17], eight [12], and nine PCRs, respectively [15]. To the best of our knowledge, this is the first study to perform the highest number of PCRs for identifying as many as 13 PMQR genes in NTS that detected six CIP-nonsusceptible isolates harboring only PMQR genes without mutations in the QRDR. A recent review article concluded that PMQR genes generally lead to only low-level quinolone resistance that does not exceed the clinical breakpoint [11]. However, we found that the presence of the three PMQR genes *oqxA*, *oqxB*, and *qnrS* in two isolates and the single gene *qnrS* in one isolate exhibited a phenotype of CIP resistance with a higher MIC of 1–2 μg/mL. Our new finding indicated that PMQR alone without QRDR mutations conferred a considerable level of quinolone resistance exceeding the clinical breakpoint.

PMQR genes usually conferred decreased susceptibility to FQs, but accelerated the selection of mutants with high quinolone resistance [31], and their actual prevalence varied widely from <1% to >50% depending on resistance mechanisms and bacterial species [11]. A recent study using WGS or PCR detected PMQR genes *qnrB*, *qnrS*, and *qnrA* in 94% of 72 CIP-intermediate but nalidixic-susceptible NTS isolates [15]. In a Finnish study, *qnrS* and *qnrA* were the only two PMQR genes detected in CIP-nonsusceptible *S. enterica* strains [26]. Similarly, *qnrS*, *qnrA*, and *qnrB* were the three most common PMQR genes of 34 *S. enterica* strains in South Korea [17]. Apart from these three studies, our study and another study conducted in Taiwan both demonstrated that *oqxA*, *oqxB*, and *qnrS* were the three most common PMQR genes detected in quinolone-nonsusceptible NTS isolates; eleven NTS isolates with *oqxAB* in our study were all CIP resistant with MICs of 1–2 μg/mL, whereas a plasmid carrying *oqxAB* was identified in nine CIP-resistant *Salmonella* isolates with no mutation in *gyrA* and an MIC of 2–4 μg/mL [12]. The acquisition of an IncHI2-type plasmid harboring *oqxAB* upregulates the chromosomal efflux pump genes *acrB*, *acrA*, *tolC*, and *yceE* that enable the survival of *S*. Typhimurium under the lethal concentrations of CIP [32]. The simultaneous existence of both *oqxAB* and *aac*(6′)-*Ib*-*cr* causing a 4-fold increase in the MIC or *oqxAB* and a single *gyrA* mutation was sufficient to develop CIP resistance (MIC: 1 μg/mL) [33]. In addition, 98% of *oqxAB*-positive and <60% of *oqxAB*-negative *S*. Typhimurium strains harbored mutations in *gyrA* or *parC* [33]. By contrast, our study results revealed that 75% of *oqxAB*-positive and 85.7% of *oqxAB*-negative NTS isolates harbored mutations in *gyrA* or *parC*. Therefore, *oqxA* and *oqxB* were determined as predominant PMQR genes with geographical characteristics in Taiwan.

PMQR genes play an important role in the CIP nonsusceptibility of NTS. In *E. coli*, *aac*(6′)-*Ib*-*cr* itself resulted in low-level CIP resistance, but could act additively in *qnrA*-bearing plasmids to generate high-level CIP resistance [34]. In our study, only three CIP-nonsusceptible NTS isolates harbored *aac*(6′)-*Ib*-*cr* that coexisted with either the QRDR mutation Asp87Asn in *gyrA* or the PMQR gene *qnrS*/*qnrB*, indicating a subordinate role of *aac*(6′)-*Ib*-*cr* in CIP resistance. By contrast, CIP-resistant *Salmonella* Litchfield isolates with a MIC of 1 μg/mL harbored *aac*(6′)-*Ib*-*cr* and *qnrB* [15], suggesting that additional factors responsible for the tuning of CIP resistance. In previous studies, *qnr* genes were frequently associated with CIP nonsusceptibility and low-level resistance in *Salmonella*, including *qnrS1* in *Salmonella* isolates with MICs of 0.125–0.25 μg/mL [17], *qnrD* in one CIP-nonsusceptible *Salmonella* isolate with a MIC of 0.5 μg/mL, *qnrS* in three CIP-resistant *Salmonella* isolates with MICs of 1–4 μg/mL [12], and *qnrS1* alone to reduce susceptibility to CIP MICs of 0.25–1 μg/mL in the absence of *gyrA* mutation [35]. Acquisition of *qnrS1* is often associated with a single *gyrA* mutation in *S.* Typhimurium, and combination of *qnrS1* and other PMQR genes is observed in other serotypes [36]. In our study, we found one CIP-resistant NTS isolate (MIC: 1 μg/mL) with *qnrS* alone in the absence of a QRDR mutation and other PMQR genes, suggesting the crucial role of *qnrS* in CIP resistance. WGS detected *qnrB19* only but no QRDR mutations in CIP-resistant *S*. *enterica* serovar Isangi nonhuman isolates [37]. In our study, the co-existence of *qnrB* and *aac*(6′)-*Ib-cr* as well as *qnrB* with mutations in *parC* contributed to low-level CIP nonsusceptibility. Overall, PMQR genes confer CIP resistance alone or synergistically with other genetic determinants.

## 4. Materials and Methods

### 4.1. Bacterial Strains and Serotyping

A total of 39 CIP-nonsusceptible NTS clinical isolates were obtained from different regions of Taiwan between 2010 and 2016, including 34 (7%) of 488 NTS isolates from northern, central, southern, and eastern Taiwan collected in the Taiwan Surveillance of Antibiotic Resistance from NHRI during 2010 to 2016, and 5 NTS isolates from TMU-SHH during 2012 to 2016 (Table 5). The acquisition and utilization of these clinical isolates were approved by the Joint Institutional Review Board of TMU (TMU-JIRB No. N201602020) and the Biosafety Committee of Taipei Medical University Shuang Ho Hospital (No. BSL-2-0048). WGS was performed using MiSeq (Illumina, San Diego, CA, USA) in 9 of the 39 isolates, and serotypes were obtained through multilocus sequence typing.

### 4.2. Antibiotic Susceptibility Test

The antibiotic susceptibility of CIP, ampicillin (AMP), and ceftriaxone (CRO) was determined using the disc inhibition test and by calculating their MICs according to the interpretive criteria provided in the Clinical and Laboratory Standards Institute (CLSI) guideline 2020 [38]. Antibiotic susceptibility was determined by measuring the diameters of inhibition zones and the MICs using the microdilution method for CIP and BD Phoenix (BD Biosciences, Flanklin Lakes, NJ, USA) for AMP and CRO (Table 5).

### 4.3. Searching Mutations and Genes Associated with Quinolone Resistance in Three Databases

Genetic loci associated with quinolone resistance, including genomic mutations and plasmid genes, were thoroughly searched from ResFinder (https://cge.cbs.dtu.dk/services/ResFinder/database.php, accessed on 31 May 2018), ARG-ANNOT (https://www.mediterranee-infection.com/acces-ressources/base-de-donnees/arg-annot-2/, accessed on 31 May 2018), and PubMed (https://www.ncbi.nlm.nih.gov/pubmed, accessed on 31 May 2018). A total of 12 reported genomic mutations in QRDR (Table S1) and 13 PMQR genes (*qnrA*, *qnrS*, *qnrB*, *aac*(6′)-*Ib*-*cr*, *qepA*, *qnrC*, *qnrD*, *oqxA*, *oqxB*, *qnrAS*, *qnrSM*, *qnrVP*, and *qnrVV*) were found to be associated with quinolone resistance.

### 4.4. Sequencing for the Detection of Genomic Mutations in the QRDR

Genomic DNA was isolated from the bacterial cultures of the 39 CIP-nonsusceptible NTS isolates using the bacterial genomic DNA purification kit (GeneMark, Taichung, Taiwan) according to the manufacturer’s instructions. According to the mutation profiles of *gryA*, *gyrB*, *parC*, and *parE* (Table S1), the sequences of primer pairs were designed to generate PCR amplicons containing these genomic mutations in the four QRDR genes (Figure S3). DNA fragments corresponding to the QRDR genes of these strains were amplified through PCR using the designed primer pairs (Table S3A). In the GeneAmp PCR System 2700 Thermal Cycler (Applied Biosystems, Life Technologies, Carlsbad, CA, USA), 10 ng/μL of template DNA was amplified in a 40-μL reaction solution containing 1 μM of each primer, 5 U of DreamTag DNA polymerase (Thermo Fisher Scientific, Waltham, USA), 63 μM of each deoxynucleoside triphosphate (Protech Technology Enterprise Co., Ltd., Taipei, Taiwan), and PCR buffer (Thermo Fisher Scientific, Waltham, MA, USA), with initial denaturation at 94 °C for 5 min, 35 cycles of denaturation at 94 °C for 30 s, annealing at 56 °C (*gyrB*) or 58 °C (*gyrA*, *parC*, and *parE*) for 30 s, and extension at 72 °C for 1 min, followed by the final extension at 72 °C for 7 min. Subsequently, the PCR products were purified using the gel/PCR DNA fragment extraction kit (Geneaid, New Taipei City, Taiwan) and sequenced using the ABI 3730 XL DNA Analyzer (Applied Biosystems, Life Technologies, Carlsbad, CA, USA) with the BigDye Terminator v3.1 Cycle Sequencing Kit (Applied Biosystems) for examining the reported genetic mutations of *gyrA*, *gyrB*, *parC*, and *parE* in the QRDR.

### 4.5. PCRs for the Detection of the 13 PMRQ Genes

Plasmid DNA was isolated from the bacterial cultures of the 39 CIP-nonsusceptible NTS isolates using a plasmid DNA purification kit (Protech Gene-Spin MiniPrep Purification Kit, Taipei, Taiwan) according to the manufacturer’s protocol. PCR was performed using specific primers designed with the help of BLAST (Table S3B). In the GeneAmp PCR System 2700 (Applied Biosystems), 10 ng/μL of template DNA was amplified in a 40-μL reaction solution containing 1 μM of each primer, 5 U of DreamTag DNA polymerase (Thermo Fisher Scientific), 63 μM of each deoxynucleoside triphosphate (Protech Technology Enterprise Co., Ltd., Taipei, Taiwan), and PCR buffer (Thermo Fisher Scientific), with initial denaturation at 94 °C for 5 min, 35 cycles of denaturation at 94 °C for 30 s, annealing at 50 °C (*qnrS* and *qnrB*), 55 °C (*qnrA*, *qepA*, *qnrC*, *qnrD*, *qnrAS*, *qnrVP*, and *qnrVV*) or 60 °C (*aac*(6′)*-Ib-cr*, *oqxA*, *oqxB*, and *qnrSM*) for 30 s, followed by extension at 72 °C for 1 min and the final extension at 72 °C for 7 min. Subsequently, 6 μL of the amplified PCR product was electrophoresed through a 1.3% agarose gel containing 1× of SYBR Safe DNA Gel Stain (Invitrogen, Life Technologies, Carlsbad, CA, USA) in 1× TBE buffer. Gel electrophoresis was performed at 100 V for 30 min to separate the genes by their molecular weights, and the PCR products were visualized under ultraviolet light using the AlphaImager Mini Imaging System (ProteinSimple, San Jose, CA, USA). In addition to the available isolates (*qnrS* in C26; *oqxA*, *oqxB*, and *qnrB* in C29; and *qnrD*, *qnrA* and *aac*(6′)-*Ib*-*cr* in another two CIP-susceptible isolates) carrying these seven PMQR genes, we generated one recombinant *S.* Typhimurium SL1344 strain carrying a synthetic DNA fragment (synthesized by BioBasic, Markham, ON, Canada), containing parts of sequences from *qepA*, *qnrC*, *qnrAS*, *qnrSM*, *qnrVP*, and *qnrVV* (Figure S4) and used it as the positive control in the PCR detection of these genes.

### 4.6. Statistical Analysis

The associations between categorical variables in QRDR mutation numbers and different ranges of MICs were analyzed using the chi-square test and Fisher’s exact test. Statistical analysis was performed using Statistical Package for Social Science (SPSS) version 21.0. A *p* value of <0.05 was considered statistically significant.

## 5. Conclusions

This present study demonstrated that QRDR mutations, although not predominant, were more common than PMQR genes in CIP-nonsusceptible NTS in Taiwan. Only two genetic loci, Thr57Ser in *parC* and Ser83Phe in *gyrA*, were detected in more than 50% of ciprofloxacin resistant NTS isolates. The grouping analysis showed significant positive association between QRDR mutation numbers and MICs (*p* < 0.001). Double QRDR mutations in *gyrA* and *parC* determined high CIP resistance with MICs of ≥4 μg/mL, whereas PMQR genes contributed to intermediate to low CIP resistance with MICs of 0.25–2 μg/mL, thus providing insights into mechanisms underlying CIP resistance.

## Figures and Tables

**Table 1:** Genomic point mutations of the four QRDR genes and presence of PMQR genes in the 39 CIP-nonsusceptible NTS isolates.

Isolate ID	QRDR Mutations												PMQR Genes				
	*gyrA*					*parC*					*parE*		*aac*(6′)-*Ib*-*cr*	*oqxA*	*oqxB*	*qnrB*	*qnrS*
	C248T (Ser83Phe)	C248A (Ser83Tyr)	G259A (Asp87Asn)	A260G (Asp87Gly)		C170G (Thr57Ser)	A238C (Ser80Arg)	G239T (Ser80IIe)	G250A (Glu84Lys)		T1372C (Ser458Pro)						
C01	+	–	–	+		+	+	–	–		+		–	–	–	–	–
C02	+	–	–	+		+	+	–	–		-		–	–	–	–	–
C03	+	–	–	+		+	+	–	–		+		–	–	–	–	–
C04	+	–	–	+		+	+	–	–		+		–	–	–	–	–
C05	+	–	+	–		+	–	–	+		–		–	–	–	–	–
C06	+	–	–	+		+	+	–	–		+		–	–	–	–	–
C07	+	–	–	+		+	+	–	–		+		–	–	–	–	–
C08	–	+	–	–		–	–	–	–		–		–	+	+	–	–
C09	–	+	–	–		–	–	–	–		–		–	+	+	–	–
C10	–	+	–	–		–	–	–	–		–		–	+	+	–	–
C11	+	–	+	–		+	–	+	–		–		–	–	–	–	–
C12	+	–	+	–		+	–	+	–		–		–	–	–	–	–
C13	–	+	–	–		–	–	–	–		–		–	+	+	–	–
C14	–	+	–	–		–	–	–	–		–		–	+	+	–	–
C15	+	–	+	–		+	–	+	–		–		–	–	–	–	–
C16	+	–	–	+		+	+	–	–		+		–	–	–	–	–
C17	–	+	–	–		–	–	–	–		–		–	+	+	–	–
C18	+	–	–	+		+	+	–	–		+		–	–	–	–	–
C19	+	–	+	–		+	–	+	–		–		–	–	–	–	–
C20	–	+	–	–		–	–	–	–		–		–	+	+	–	–
C21	+	–	–	+		+	+	–	–		+		–	–	–	–	–
C22	+	–	+	–		+	–	+	–		–		–	–	–	–	–
C23	–	+	–	–		–	–	–	–		–		–	+	+	–	–
C24	+	–	+	–		+	–	+	–		–		–	–	–	–	–
C25	+	–	+	–		+	–	+	–		–		–	–	–	–	–
C26	–	–	–	–		–	–	–	–		–		–	+	+	–	+
C27	–	–	–	–		–	–	–	–		–		–	+	+	–	+
C28	+	–	+	–		+	–	+	–		–		–	–	–	–	–
C29	–	–	+	–		–	–	–	–		–		+	+	+	–	–
C30	–	–	–	–		–	–	–	–		–		+	–	–	–	+
C31	+	–	–	–		+	–	–	–		–		–	–	–	–	–
C32	–	–	–	–		–	–	–	–		–		+	–	–	–	+
C33	–	+	–	–		+	–	–	–		–		–	–	–	–	+
C34	–	–	–	–		–	–	–	–		–		–	–	–	–	+
C35	–	+	–	–		+	–	–	–		–		–	–	–	–	+
C36	–	–	–	–		–	–	–	–		–		+	–	–	+	–
C37	+	–	–	–		+	–	–	–		–		–	–	–	–	–
C38	–	–	–	–		+	–	–	–		–		–	–	–	+	–
C39	+	–	–	–		–	–	–	–		–		–	–	–	–	–
Total: 39	21	10	10	9		23	9	8	1		8		4	11	11	2	7
Percentage	53.8%	25.6%	25.6%	23.0%		58.9%	23.0%	20.5%	2.6%		20.5%		10.3%	28.2%	28.2%	5.1%	18.0%

**Table 2:** Grouping of the ciprofloxacin nonsusceptibility profiles according to MICs and combination types of QRDR mutations and PMQR genes in the 39 NTS isolates.

Group (Isolate No.)	MIC (μg/mL)/Isolate ID	Combination Type	QRDR Mutations			PMQR Genes
			*gyrA*	*parC*	*parE*	
1 (n = 8)	32/C01, C03, C04, C06, C07, C16, C18, C21	I	Ser83Phe Asp87Gly	Thr57Ser Ser80Arg	Ser458Pro	–
2 (n = 10)	16/C12, C15, C28 8/C19, C22, C24, C25 4/C11	II	Ser83Phe Asp87Asn	Thr57Ser Ser80IIe	–	–
	8/C05	III	Ser83Phe Asp87Asn	Thr57Ser Glu84Lys	–	–
	8/C02	IV	Ser83Phe Asp87Gly	Thr57Ser Ser80Arg	–	–
3 (n = 14)	2/C29	V	Asp87Asn	–	–	*aac*(6′)-*Ib*-*cr* *oqxA*, *oqxB*
	2/C26, C27	VI	–	–	–	*oqxA*, *oqxB*, *qnrS*
	1/C33, C35	VII	Ser83Tyr	Thr57Ser	–	*qnrS*
	1/C08, C09, C10, C13, C14, C17, C20, C23	VIII	Ser83Tyr	–	–	*oqxA*, *oqxB*
	1/C34	IX	–	–	–	*qnrS*
4 (n = 7)	0.5/C31, C37	X	Ser83Phe	Thr57Ser	–	–
	0.5/36	XI	–	–	–	*aac*(6′)-*Ib*-*cr* *qnrB*
	0.5/C32 0.25/C30	XII	–	–	–	*aac*(6′)-*Ib*-*cr* *qnrS*
	0.25/C39	XIII	Ser83Phe	–	–	–
	0.25/C38	XIV	–	Thr57Ser	–	*qnrB*

**Table 3:** The number distribution of different MICs among four groups.

Grouping by MICs	No. of Isolates	Number of Different MICs (μg/mL)							
		0.25	0.5	1	2	4	8	16	32
1 (32 μg/mL)	8	0	0	0	0	0	0	0	8
2 (4–16 μg/mL)	10	0	0	0	0	1	6	3	0
3 (1–2 μg/mL)	14	0	0	11	3	0	0	0	0
4 (0.25–0.5 μg/mL)	7	3	4	0	0	0	0	0	0
Total	39	3	4	11	3	1	6	3	8

**Table 4:** Cross tabulation of QRDR mutation numbers and MIC groups.

Grouping by QRDR Mutation No.	Groups (MICs)			
	1 (32 μg/mL)	2 (4–16 μg/mL)	3 (1–2 μg/mL)	4 (0.25–0.5 μg/mL)
1 (5 mutations)				
Case No. (%)	8 (100) *	0 (0)	0 (0)	0 (0)
2 (4 mutations)				
Case No. (%)	0 (0)	10 (100) *	0 (0)	0 (0)
3 (0–3 mutations)				
Case No. (%)	0 (0)	0 (0)	14 (100) *	7 (100) *
Total Case No.	8	10	14	7

* *p* < 0.001, Fisher’s exact test.

**Table 5:** The 39 clinical isolates of CIP-nonsusceptible NTS and their antibiotic susceptibility to three antibiotics according to the CLSI guideline 2020.

Isolate ID	Year	Region	Serotype	Disc Inhibition Test			MIC (μg/mL)		
				CIP *	AMP ^†^	CRO ^‡^	CIP *	AMP ^†^	CRO ^‡^
C01	1998	S	–	R	R	S	32	>16	≤1
C02	1998	C	Schwarzengrund	R	R	S	8	>16	≤1
C03	1998	C	Schwarzengrund	R	R	S	32	>16	≤1
C04	1998	S	–	R	R	S	32	>16	≤1
C05	1998	S	–	R	S	S	8	≤4	≤1
C06	1998	S	–	R	S	S	32	≤4	≤1
C07	2000	C	Schwarzengrund	R	R	S	32	>16	≤1
C08	2000	E	–	R	R	S	1	>16	≤1
C09	2000	E	–	R	R	S	1	>16	≤1
C10	2000	E	–	R	R	S	1	>16	≤1
C11	2000	E	–	R	R	S	8	>16	≤1
C12	2000	E	–	R	R	S	16	>16	≤1
C13	2000	N	–	R	R	S	1	>16	≤1
C14	2000	C	Typhimurium	I	R	S	1	>16	≤1
C15	2000	S	–	R	R	S	16	>16	≤1
C16	2002	S	–	R	R	S	32	>16	≤1
C17	2002	S	–	R	S	S	1	≤4	1
C18	2002	N	–	R	R	R	32	>16	8
C19	2002	E	–	R	R	S	32	>16	≤1
C20	2002	E	–	R	R	S	1	>16	≤1
C21	2002	E	–	R	R	S	32	>16	≤1
C22	2002	E	–	R	R	S	8	>16	≤1
C23	2002	C	–	R	S	S	1	≤4	≤1
C24	2002	N	–	R	R	S	8	>16	≤1
C25	2002	C	Choleraesuis	R	R	S	8	>16	≤1
C26	2010	S	–	R	S	S	2	≤4	≤1
C27	2012	E	–	R	R	S	2	>16	≤1
C28	2012	S	–	R	R	S	16	>16	≤1
C29	2012	C	Typhimurium	R	R	S	2	>16	≤1
C30	2012	C	Typhimurium	I	R	S	0.25	>16	≤1
C31	2012	C	–	I	R	S	0.5	>16	≤1
C32	2012	C	–	I	R	S	0.5	>16	≤1
C33	2014	C	Enteritidis	R	R	S	1	>16	≤1
C34	2014	C	–	R	R	S	1	>16	≤1
C35	2016	N (SHH)	–	I	R	S	0.5	>16	≤1
C36	2015	N (SHH)	–	I	R	R	0.5	>16	>32
C37	2014	N (SHH)	Albany	I	R	S	0.5	>16	≤1
C38	2016	N (SHH)	–	I	R	S	0.25	>16	≤1
C39	2013	N (SHH)	–	I	R	S	0.25	>16	≤1

N: northern, C: central, S: south, E: eastern, SHH: Shuang Ho Hospital; R: resistant, I: intermediate; –: not done; Amp: ampicillin, CIP: ciprofloxacin, CRO: ceftriaxone; * Disc diameters: ≥31 mm (S), 21–30 mm (I), and ≤20 mm (R) and MIC: ≤0.06 μg/mL (S), 0.12–0.5 μg/mL (I), and ≥1 μg/mL (R) for CIP-susceptibility; ^†^ Disc diameters: ≥17 mm (S), 14–16 mm (I), and ≤13 mm (R) and MIC: ≤8 μg/mL (S), 16 μg/mL (I), and ≥32 μg/mL (R) for AMP-susceptibility; ^‡^ Disc diameters: ≥23 mm (S), 20–22 mm (I), and ≤19 mm (R) and MIC: ≤1 μg/mL (S), 2 μg/mL (I), and ≥4 μg/mL (R) for CRO-susceptibility.

## Data Availability

Sequences of the 4 QRDR genes in the 39 NTS clinical isolates related to this article can be found, in the online version, at DOI: 10.5281/zenodo.5593175.

## Supplementary Materials

The following are available online at https://www.mdpi.com/article/10.3390/antibiotics10111383/s1, Figure S1: PCR detection of the PMQR genes *aac*(6′)-*Ib*-*cr* (A), *oqxA* (B), *oqxB* (C), *qnrB* (D), and *qnrS* (E) in the 39 CIP-nonsusceptible NTS isolates (white arrows indicate the presence of PMQR genes; bp: base pair, MK: marker, SL: *Salmonella* Typhimurium SL1344), Figure S2: PCR detection of the PMQR genes *qepA* (A), *qnrA* (B), *qnrC* (C), *qnrD* (D), *qnrAS* (E), *qnrSM* (F), *qnrVP* (G), and *qnrVV* (H) in the 39 CIP-nonsusceptible NTS isolates (bp: base pair, MK: marker, SL: *Salmonella* Typhimurium SL1344), Figure S3: Schematic of the generation of PCR amplicons comprising the reported mutations in the four QRDR genes of CIP-nonsusceptible NTS clinical isolates, Figure S4: Design map of the synthesized DNA fragment containing parts of the selected six PMQR gene sequences, ligated with plasmid pUC57 using the restriction enzymes *Hin* dIII and *Mlu* I for cloning into *S*. Typhimurium SL1344 as the recombinant strain, as the positive control of the six PMQR genes, Table S1: Mutational profiles of *gryA*, *gyrB*, *parC*, and *parE* related to CIP resistance, Table S2: Distribution of QRDR mutations and PMQR genes in the 39 CIP-nonsusceptible NTS isolates, Table S3: Sequences of primer pairs for PCR amplicons for the four QRDR genes *gyrA*, *gyrB*, *parC*, and *parE* (A) and PCR detection of the 13 reported PMQR genes (B). References [39,40,41,42,43,44,45,46,47,48] are cited in the Supplementary Materials.
