# Supplementary material for: Genotypic Diversity of Ciprofloxacin Nonsusceptibility and Its Relationship with Minimum Inhibitory Concentrations in Nontyphoidal Salmonella Clinical Isolates in Taiwan
Source: Antibiotics (Basel). 2021 Nov 11;10(11):1383. doi: 10.3390/antibiotics10111383 (PMC8614936; doi:10.3390/antibiotics10111383)
Supplement: Supplementary file 1 [file antibiotics-10-01383-s001.zip › Table S1. Mutational profiles of gryA, gyrB, parC, and parE.pdf]

Table S1. Mutational profiles of *gryA*, *gyrB*, *parC*, and *parE* related to CIP resistance

No.	Gene	Mutational site	Mutant construction or restoration	Origin	Reference
1	*gyrA*	Ser83Phe	No	*Salmonella*	Reyna F, et al. Antimicrob Agents Chemother, 1995. 39(7):1621-3. [39]
2	*gyrA*	Ser83Phe	Yes	*Salmonella*	Turner AK, et al. J Antimicrob Chemother, 2006. 58(4):733-40. [40]
3	*gyrA*	Ser83Tyr	No	*Salmonella*	Reyna F, et al. Antimicrob Agents Chemother, 1995. 39(7):1621-3. [39]
4	*gyrA*	Ser83Tyr	Yes	*Salmonella*	Turner AK, et al. J Antimicrob Chemother, 2006. 58(4):733-40. [40]
5	*gyrA*	Ser83Ala	Yes	*E. coli*	Barnard FM, et al. Antimicrob Agents Chemother, 2001. 45(7):1994-2000. [41]
6	*gyrA*	Ser83Leu	No	*E. coli*	Cesaro A, et al. J Antimicrob Chemother, 2008. 61(5):1007-15. [42]
7	*gyrA*	Ser83stop	No	*E. coli*	Cesaro A, et al. J Antimicrob Chemother, 2008. 61(5):1007-15. [42]
8	*gyrA*	Asp87Tyr	No	*Salmonella*	Reyna F, et al. Antimicrob Agents Chemother, 1995. 39(7):1621-3. [39]
9	*gyrA*	Asp87Tyr	Yes	*Salmonella*	Turner AK, et al. J Antimicrob Chemother, 2006. 58(4):733-40. [40]
10	*gyrA*	Asp87Asn	No	*Salmonella*	Reyna F, et al. Antimicrob Agents Chemother, 1995. 39(7):1621-3. [39]
11	*gyrA*	Asp87Asn	Yes	*Salmonella*	Turner AK, et al. J Antimicrob Chemother, 2006. 58(4):733-40. [40]
12	*gyrA*	Asp87Ala	Yes	*E. coli*	Barnard FM, et al. Antimicrob Agents Chemother, 2001. 45(7):1994-2000. [41]
13	*gyrA*	Asp87Gly	No	*Salmonella*	Lin CC, et al. J Food Prot, 2009. 72(1):14-20. [43]
14	*gyrA*	Asp87Gly	No	*Pseudomonas*	Yonezawa M, et al. Antimicrob Agents Chemother, 1995. 39(9):1970-2. [44]
15	*gyrA*	Ala67Pro+Gly 81Ser	No	*Salmonella*	Reyna F, et al. Antimicrob Agents Chemother, 1995. 39(7):1621-3. [39]
16	*gyrA*	Asp82Asn	No	*E. coli*	Cesaro A, et al. J Antimicrob Chemother, 2008. 61(5):1007-15. [42]
17	*gyrB*	Glu466Asp	Yes	*Salmonella*	Al-Emran HM, et al. Clin Infect Dis, 2016. 62 Suppl 1:S47-9. [45]
18	*gyrB*	Insertion of Glu at 465	No	*E. coli*	Cesaro A, et al. J Antimicrob Chemother, 2008. 61(5):1007-15. [42]
19	*gyrB*	Tyr420Cys	No	*Salmonella*	Eaves DJ, et al. Antimicrob Agents Chemother, 2004. 48(10):4012-4015. [46]
20	*gyrB*	Arg437Leu	No	*Salmonella*	Eaves DJ, et al. Antimicrob Agents Chemother, 2004. 48(10):4012-4015. [46]
21	*parC*	Thr57Ser	No	*Salmonella*	Eaves DJ, et al. Antimicrob Agents Chemother, 2004. 48(10):4012-4015. [46]

22	*parC*	Thr66Ile	No	*Salmonella*	Eaves DJ, et al. Antimicrob Agents Chemother, 2004. 48(10):4012-4015. [46]
23	*parC*	Ser80Arg	No	*Salmonella*	Ling JM, et al. Antimicrob Agents Chemother, 2003. 47(11):3567-73. [27]
24	*parC*	Ser80IIe	No	*E. coli*	Saenz Y, et al. J Antimicrob Chemother, 2003. 51(4):1001-5. [47]
25	*parC*	Glu84Gly	No	*E. coli*	Saenz Y, et al. J Antimicrob Chemother, 2003. 51(4):1001-5. [47]
26	*parC*	Glu84Lys	Yes	*Salmonella*	Turner AK, et al. J Antimicrob Chemother, 2006. 58(4):733-40. [40]
27	*parE*	Glu453Gly	No	*Salmonella*	Eaves DJ, et al. Antimicrob Agents Chemother, 2004. 48(10):4012-4015. [46]
28	*parE*	Ala498Thr	No	*Salmonella*	Eaves DJ, et al. Antimicrob Agents Chemother, 2004. 48(10):4012-4015. [46]
29	*parE*	Val512Gly	No	*Salmonella*	Eaves DJ, et al. Antimicrob Agents Chemother, 2004. 48(10):4012-4015. [46]
30	*parE*	Ser518Cys	No	*Salmonella*	Eaves DJ, et al. Antimicrob Agents Chemother, 2004. 48(10):4012-4015. [46]
31	*parE*	Ser458Pro	No	*Salmonella*	Ling JM, et al. Antimicrob Agents Chemother, 2003. 47(11):3567-73. [27]
32	*parE*	Val461Gly	Yes (no function)	*Salmonella*	O’Regan E, et al. Antimicrob Agents Chemother, 2009. 53(3):1080-7. [48]
