# Supplementary material for: Genotypic Diversity of Ciprofloxacin Nonsusceptibility and Its Relationship with Minimum Inhibitory Concentrations in Nontyphoidal Salmonella Clinical Isolates in Taiwan
Source: Antibiotics (Basel). 2021 Nov 11;10(11):1383. doi: 10.3390/antibiotics10111383 (PMC8614936; doi:10.3390/antibiotics10111383)
Supplement: Supplementary file 1 [file antibiotics-10-01383-s001.zip › Table S2. Distribution of QRDR mutations and PMQR genes.pdf]

Table S2. Distribution of QRDR mutations and PMQR genes in the 39 CIP-nonsusceptible NTS isolates

Isolate ID	Both QRDR mutations(s) and PMQR gene(s)	QRDR mutation(s) only	Any QRDR mutation(s)	PMQR gene(s) only	Any PMQR gene(s)	Neither QRDR mutation nor PMQR gene
C01	–	+	+	–	–	–
C02	–	+	+	–	–	–
C03	–	+	+	–	–	–
C04	–	+	+	–	–	–
C05	–	+	+	–	–	–
C06	–	+	+	–	–	–
C07	–	+	+	–	–	–
C08	+	–	+	–	+	–
C09	+	–	+	–	+	–
C10	+	–	+	–	+	–
C11	–	+	+	–	–	–
C12	–	+	+	–	–	–
C13	+	–	+	–	+	–
C14	+	–	+	–	+	–
C15	–	+	+	–	–	–
C16	–	+	+	–	–	–
C17	+	–	+	–	+	–
C18	–	+	+	–	–	–
C19	–	+	+	–	–	–
C20	+	–	+	–	+	–
C21	–	+	+	–	–	–
C22	–	+	+	–	–	–
C23	+	–	+	–	+	–
C24	–	+	+	–	–	–
C25	–	+	+	–	–	–
C26	–	–	–	+	+	–
C27	–	–	–	+	+	–
C28	–	+	+	–	–	–
C29	+	–	+	–	+	–
C30	–	–	–	+	+	–
C31	–	+	+	–		–
C32	–	–	–	+	+	–
C33	+	–	+	–	+	–
C34	–	–	–	+	+	–
C35	+	–	+	–	+	–
C36	–	–	–	+	+	–
C37	–	+	+	–	–	–
C38	+	–	+	–	+	–
C39	–	+	+	–	–	–
Total: 39	12	21	33	6	18	0
Percentage	30.8%	53.8%	84.6%	15.4%	46.2%	0.0%
