# Supplementary material for: Genotypic Diversity of Ciprofloxacin Nonsusceptibility and Its Relationship with Minimum Inhibitory Concentrations in Nontyphoidal Salmonella Clinical Isolates in Taiwan
Source: Antibiotics (Basel). 2021 Nov 11;10(11):1383. doi: 10.3390/antibiotics10111383 (PMC8614936; doi:10.3390/antibiotics10111383)
Supplement: Supplementary file 1 [file antibiotics-10-01383-s001.zip › Table S3. Sequences of primer pairs for PCR amplicons.pdf]

Table S3. Sequences of primer pairs for PCR amplicons for the four QRDR genes *gyrA*, *gyrB*, *parC*, and *parE* (A) and PCR detection of the 13 reported PMQR genes (B)

Gene	Forward (5′→3′)	Reverse (5′→3′)	PCR product (bp)
A. QRDR			
gyrA	CTGGATTATGCGATGTCGGTCAT	TAGAACCGAAGTTACCCTGACCA	271
gyrB	CCCGTGAAATGACCCGTCGTAAA	ACGCTCGACAATTTCCGGCATCT	410
parC	TCATGGATCGTGCGTTG	GCGAATGACTTCGGATCA	271
parE	CGCTGCGCTTATGTGCT	CAGTACGCCCGCCTTCT	702
B. PMQR			
aac(6′)-Ib-cr	GGTATGCCCAGTCGTACGTT	AACCATGTACACGGCTGGA	295
oqxA	CTCGGCGCGATGATGCT	CCACTCTTCACGGGAGACGA	392
oqxB	TTCTCCCCCGGCGGGAAGTAC	CTCGGCCATTTTGGCGCGTA	512
qepA	CGGCACGTTGAGCCAGAA	TGGATCGCCGCGTTTT	102
qnrA	ATCCAGATCGGCAAAGGTTA	GATAAAGTTTTTCAGCAAGAGG	543
qnrB	ATCGCGAAAGTCAGAAAGG	CTTGCACCGCGAAAATCTG	160
qnrC	GCAGAATTCAGGGGTGTGAT	AACTGCTCCAAAAGCTGCTC	118
qnrD	AGGAATAGCTTGGAAGGGTG	CCTAAGGCGCTAATGTTT	107
qnrS	ACTTAAGTCGAGGTGTTT	CAATTTTGATACCTGATG	126
qnrAS	TGCGCCACGAGAAAGGTC	AACGCCTATGTATCGAGAAATG	101
qnrSM	TACACCGGCCAGAAAGTGGTCGA	TCCTTCAGCGTGGCGCCATTGAA	140
qnrVP	TGTATGTTGGCGATGAGC	GGTGTTACTGACGCGATTA	120
qnrVV	GCAGACTGTTTCGGCATTG	TGATGTAGGCGGAGCAAA	115
