# Supplementary figures and images for: Genotypic Diversity of Ciprofloxacin Nonsusceptibility and Its Relationship with Minimum Inhibitory Concentrations in Nontyphoidal Salmonella Clinical Isolates in Taiwan
Source: Antibiotics (Basel). 2021 Nov 11;10(11):1383. doi: 10.3390/antibiotics10111383 (PMC8614936; doi:10.3390/antibiotics10111383)

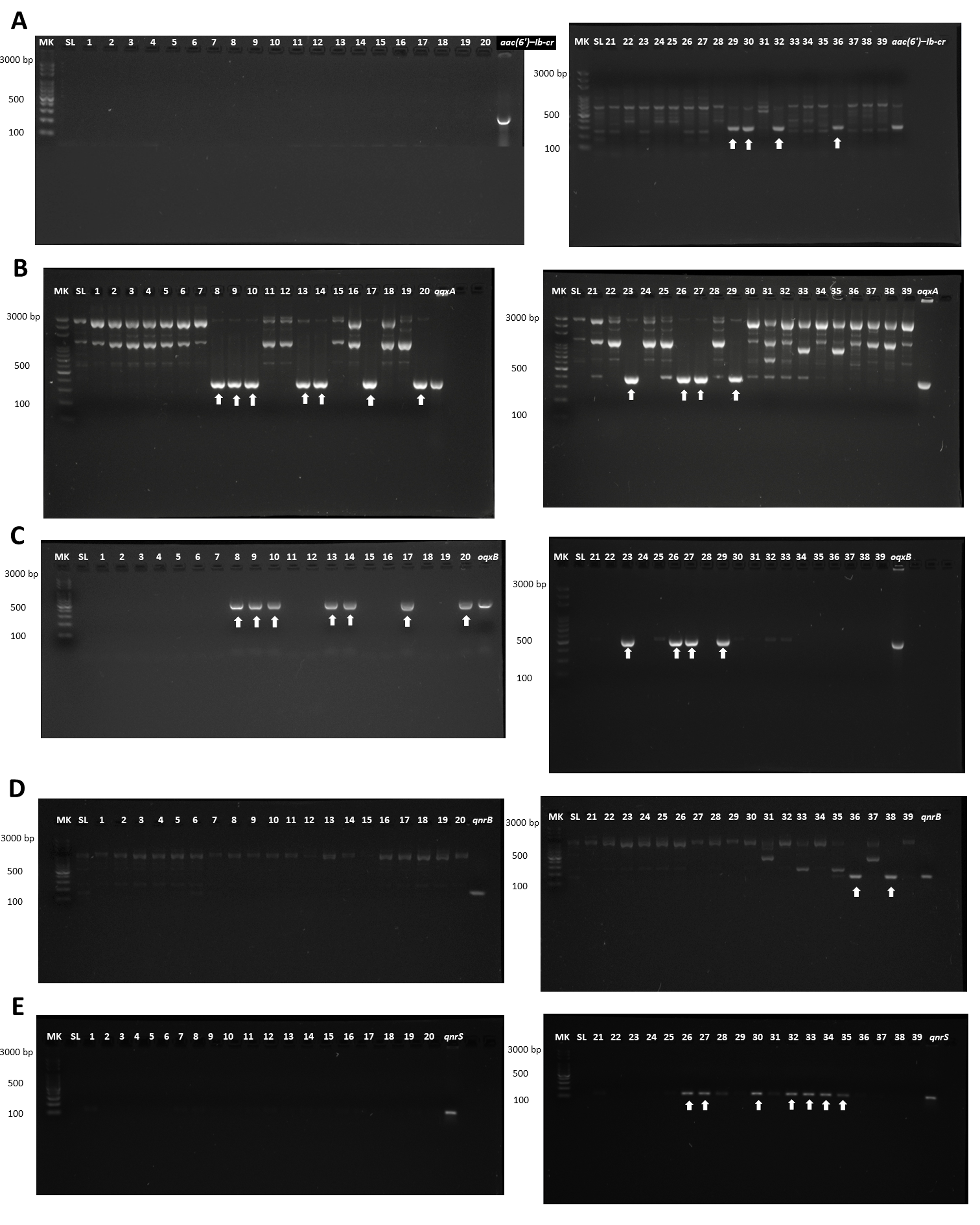

Supplement: Supplementary file 1 [file antibiotics-10-01383-s001.zip › Figure S1. PCR detection of the PMQR genes (positive).tif]

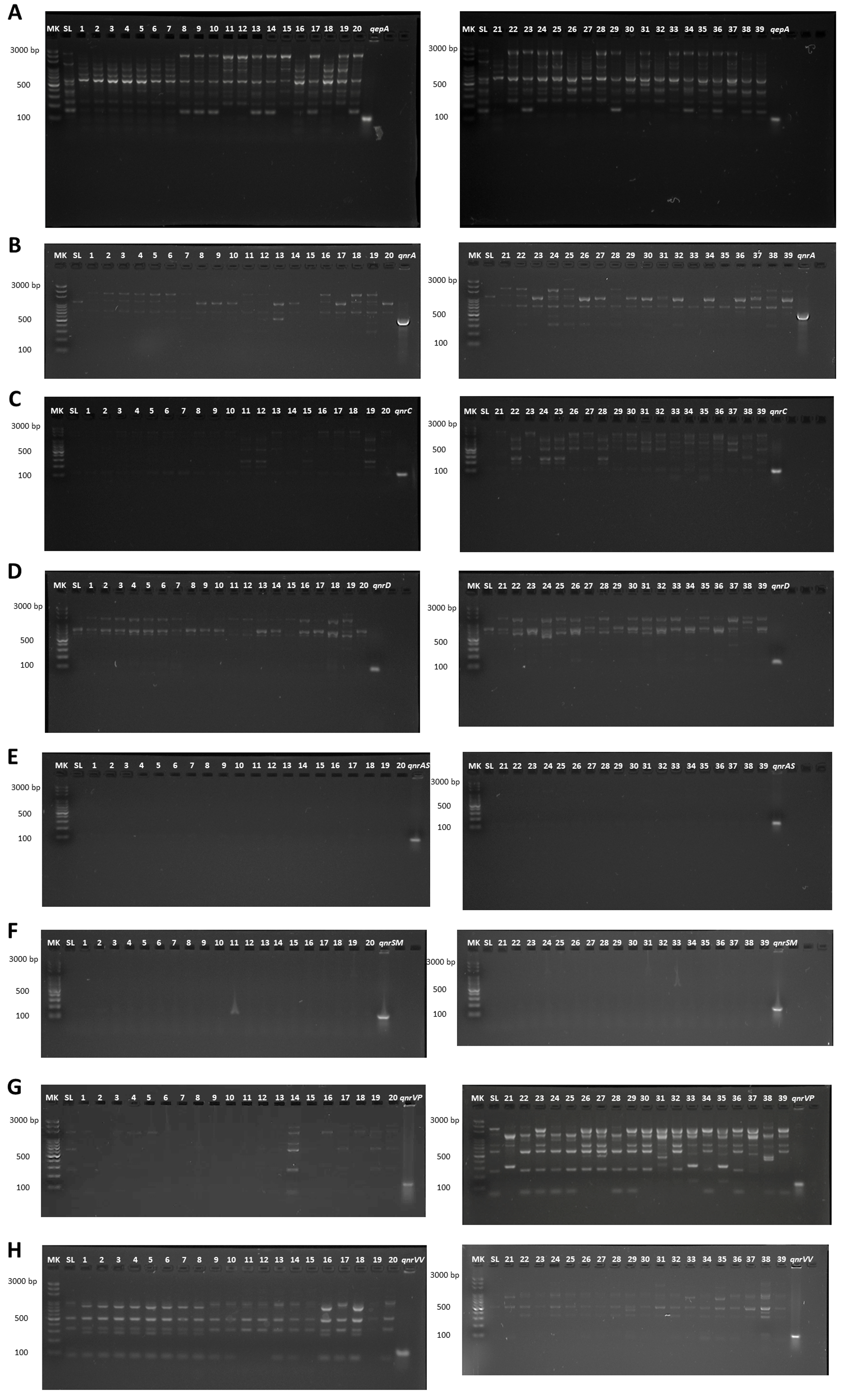

Supplement: Supplementary file 1 [file antibiotics-10-01383-s001.zip › Figure S2. PCR detection of the PMQR genes (negative).tif]

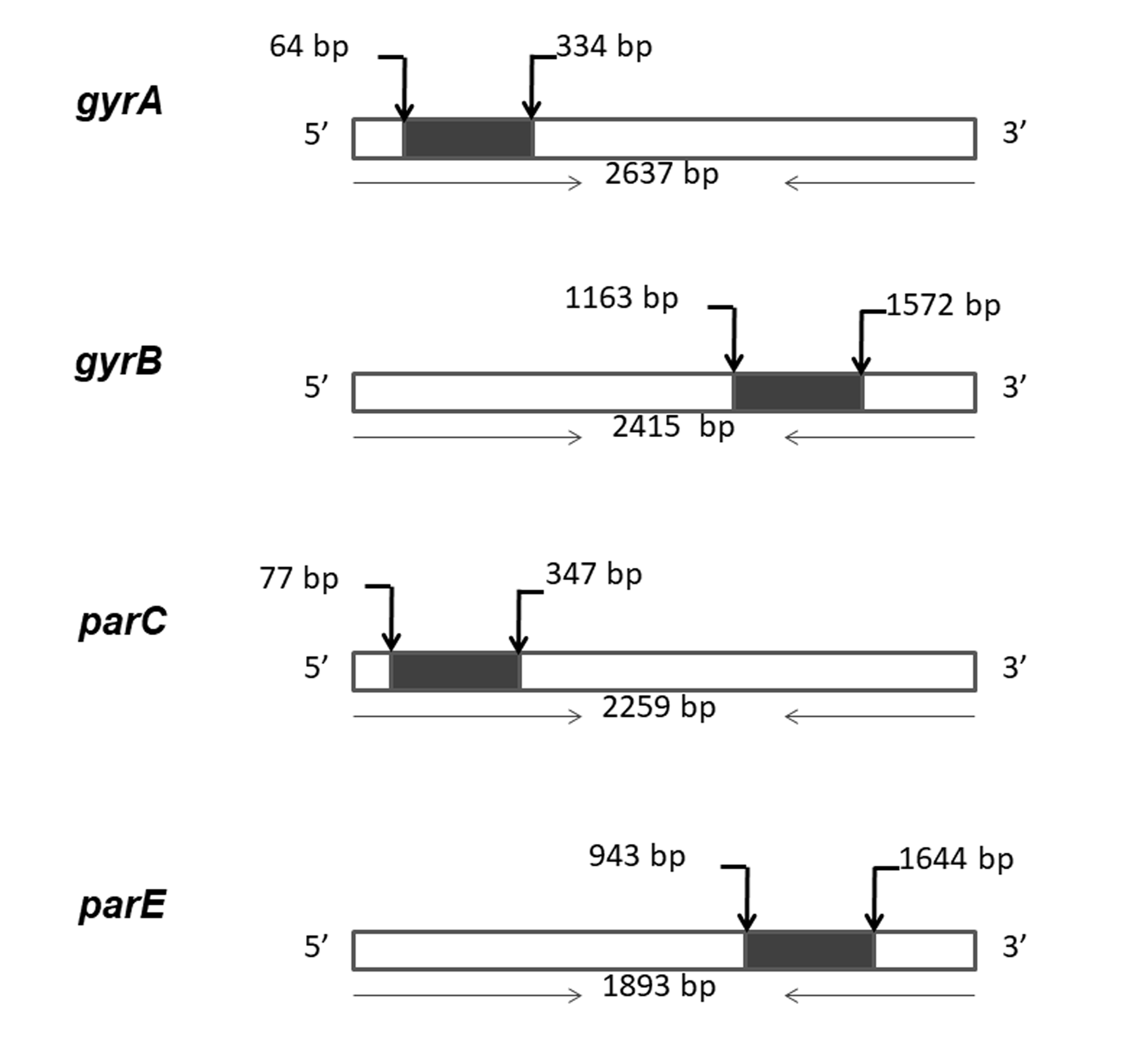

Supplement: Supplementary file 1 [file antibiotics-10-01383-s001.zip › Figure S3. Schematic of the generation of PCR amplicons.tif]

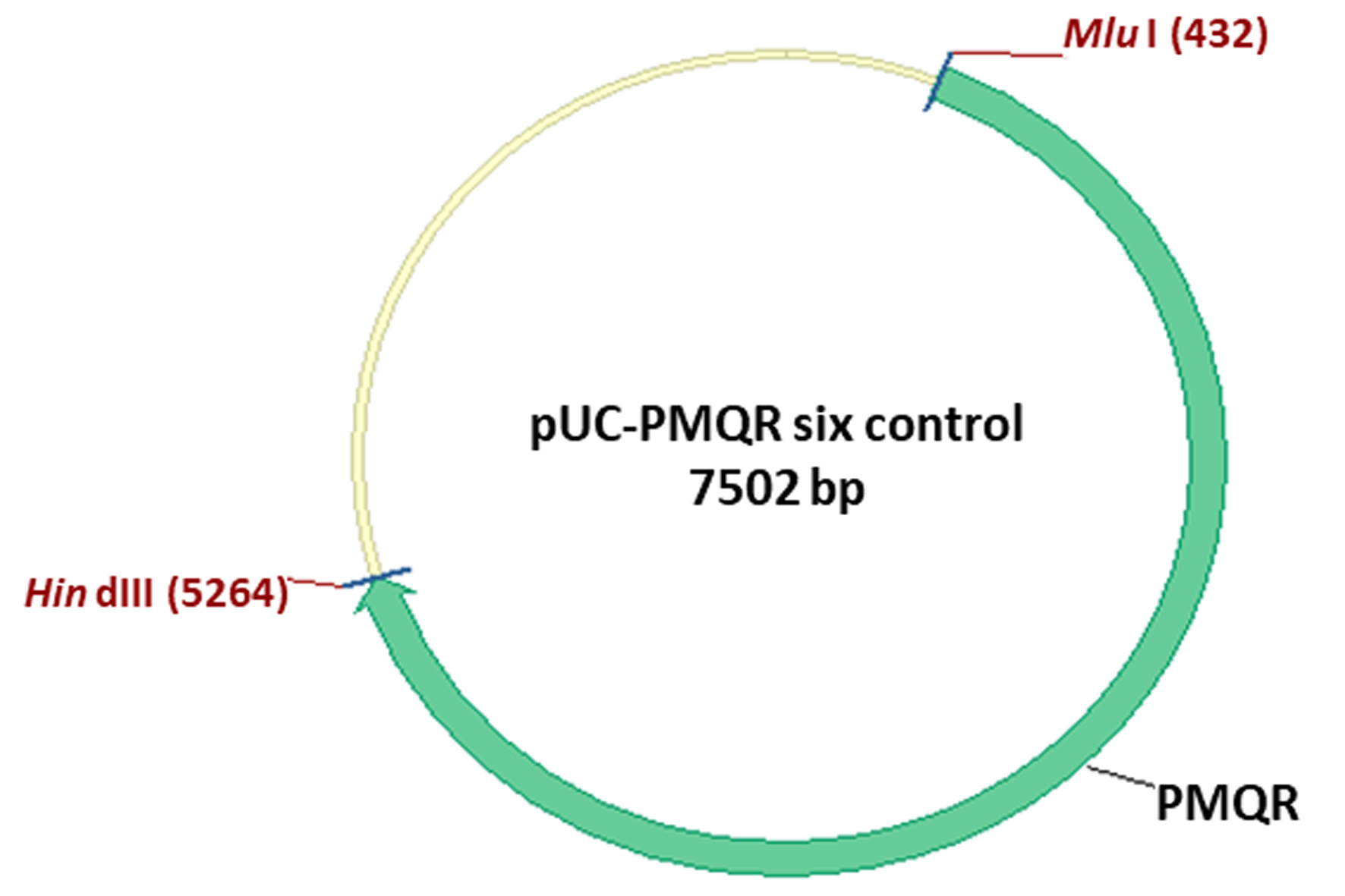

Supplement: Supplementary file 1 [file antibiotics-10-01383-s001.zip › Figure S4. Design map of the synthesized DNA frament.tif]
